# Recycling Spent LFP Batteries: From Resource Recovery to High-Value Functional Materials

**DOI:** 10.3390/molecules30173557

**Published:** 2025-08-30

**Authors:** Chang Wang, Lizhi Wang, Zixuan Fu, Fan Yin, Fangyu Zheng, Jun Wang, Fei Fang, Qiangchun Liu, Xiangkai Kong

**Affiliations:** 1 School of Physics and Electronic Information, Huaibei Normal University, Huaibei 235000, China; 2 School of Materials and Physics & Center of Mineral Resource Waste Recycling, China University of Mining and Technology, Xuzhou 221116, China

**Keywords:** spent battery, recycling, lithium iron phosphate

## Abstract

With the growing wave of end-of-life new energy vehicles, the recycling of lithium iron phosphate (LFP) batteries has become increasingly imperative. In contrast to conventional pyrometallurgical and hydrometallurgical approaches, recent efforts have shifted toward innovative recycling strategies and emerging applications for spent LFP materials. During battery operation, the irreversible oxidation of Fe^2+^ to Fe^3+^ often leads to lithium loss and performance degradation. To address this, various approaches—such as electrochemical delamination and ultrasonic separation—have been developed to efficiently detach cathode materials from current collectors, followed by thermal or wet-chemical regeneration to restore their electrochemical activity. Beyond conventional regeneration, the upcycling of spent LFP into value-added functional materials offers a sustainable pathway for resource reutilization. Notably, phosphorus extracted from LFP can be converted into slow-release fertilizers, broadening the scope of secondary applications. As the volume of spent LFP batteries continues to rise, there is an urgent need to establish an integrated recycling framework that harmonizes environmental impact, technical efficiency, and economic viability. Henceforth, this review summarizes recent advances in LFP recycling and upcycling, discusses critical challenges, and provides strategic insights for the sustainable and high-value reuse of spent LFP cathodes.

## 1. Introduction

The global energy system remains heavily dependent on fossil fuels, a reliance that continues to drive escalating environmental challenges, including rising greenhouse gas emissions, persistent air pollution, and the increasing frequency of extreme climate events [1,2,3]. In response to these pressing concerns, accelerating the transition toward carbon neutrality and the sustainable management of finite natural resources has become a central priority for governments and industries worldwide. Among various decarbonization strategies, the electrification of the transportation sector has emerged as a transformative pathway, catalyzing a rapid expansion of electric vehicle (EV) deployment and fundamentally reshaping the global mobility landscape [4,5,6].

At the core of this transition lies the lithium-ion battery (LIB), which has revolutionized energy storage systems by offering high energy density, long cycle life, and rechargeability—enabling EVs to reduce dependence on petroleum-based fuels. The exponential growth in EV adoption has, in turn, driven a massive increase in the production and deployment of LIBs. However, this unprecedented expansion has also generated a parallel and increasingly urgent challenge: the accumulation of vast quantities of end-of-life LIBs [7,8]. If improperly managed, these spent batteries pose severe environmental hazards and resource security risks. Developing sustainable, cost-effective, and environmentally sound recycling strategies is therefore critical—not only to mitigate the ecological burden of battery waste but also to recover valuable materials and support the emergence of a circular battery economy in line with long-term climate objectives.

Amid this backdrop, the deployment of LIBs in EVs has witnessed remarkable acceleration. It is reported that the global LiFePO_4_ battery market was valued at USD 8.718 billion in 2022 and is projected to reach USD 18.512 billion by 2028 [9]. Among various LIB chemistries, lithium iron phosphate (LFP) and nickel–cobalt–manganese (NCM) batteries have emerged as dominant technologies. In particular, LFP batteries have gained substantial market traction due to their intrinsic advantages, such as superior thermal and chemical stability, long cycle life, and lower production costs. These attributes make them especially attractive for large-scale applications, including commercial EVs and grid-scale energy storage. Notably, the adoption of LFP batteries in China increased dramatically, with their market share in new EV installations rising from 38.3% in 2020 to 62.4% in 2022 [10,11]. This rapid growth underscores the urgent need for recycling strategies tailored specifically to the unique structure and material composition of LFP batteries, which differ significantly from NCM and other high-value cathode systems.

Despite their enabling role in the energy transition, LIBs present an inherent contradiction: while facilitating decarbonization, their production relies heavily on non-renewable and geographically constrained metal resources. Lithium—a key component in all LIB chemistries—is a finite and strategically critical mineral, whose extraction is associated with significant environmental, economic, and geopolitical challenges [12,13]. As global demand for EVs and stationary storage systems continues to rise, the resulting surge in lithium and other critical metal prices has raised widespread concerns over long-term resource sustainability and material supply risks.

Compounding these concerns is the limited service life of LIBs, typically ranging from 5 to 8 years in EV applications. Performance degradation during use renders these batteries unsuitable for further deployment, contributing to a projected global volume of approximately 11 million metric tons of decommissioned LIBs annually by 2030 [14]. Alarmingly, current recycling rates remain relatively low, revealing systemic inefficiencies in collection, treatment, and regulatory enforcement. This situation presents a dual threat: the loss of valuable strategic resources and the risk of environmental contamination from hazardous substances released by improperly handled battery waste [15].

In the context of LFP batteries, the challenge is further exacerbated by their relatively low economic recyclability. Unlike NCM batteries, which contain high-value metals such as nickel and cobalt, the recoverable elements in LFP batteries—mainly lithium and iron—offer limited commercial incentive due to their lower market value [16,17]. This economic barrier has become a key bottleneck hindering the widespread implementation of efficient recycling solutions for LFP systems, despite their rapidly increasing presence in the retired battery stream. Therefore, there is an urgent need to develop cost-effective, scalable, and environmentally benign recycling technologies specifically designed for LFP batteries. Beyond conventional material recovery, innovative strategies such as upcycling and direct regeneration have recently emerged as promising pathways to enhance resource utilization efficiency and unlock the full circular potential of LFP-based systems.

In this review, we focus on the recycling and reuse of spent LFP batteries. We begin with a mechanistic discussion of capacity fading and performance degradation from the cathode perspective. Subsequently, we systematically evaluate the current mainstream recycling strategies—including physical pretreatment, thermal methods, hydrometallurgical processes, and emerging bioleaching technologies. We further explore representative high-value reuse approaches such as electrocatalytic and water-splitting applications. Through a comparative assessment of these technologies in terms of technical feasibility, environmental impact, and industrial scalability, we aim to provide a comprehensive understanding of the current state and future direction of LFP battery recycling. Finally, we propose an integrated recycling framework and outline key research opportunities to facilitate the sustainable recovery and value-added utilization of spent LFP cathodes within a circular economy framework.

## 2. Principles of Recycling Current Collectors

### 2.1. Failure Mechanisms of LFP Batteries

**Figure 1:**
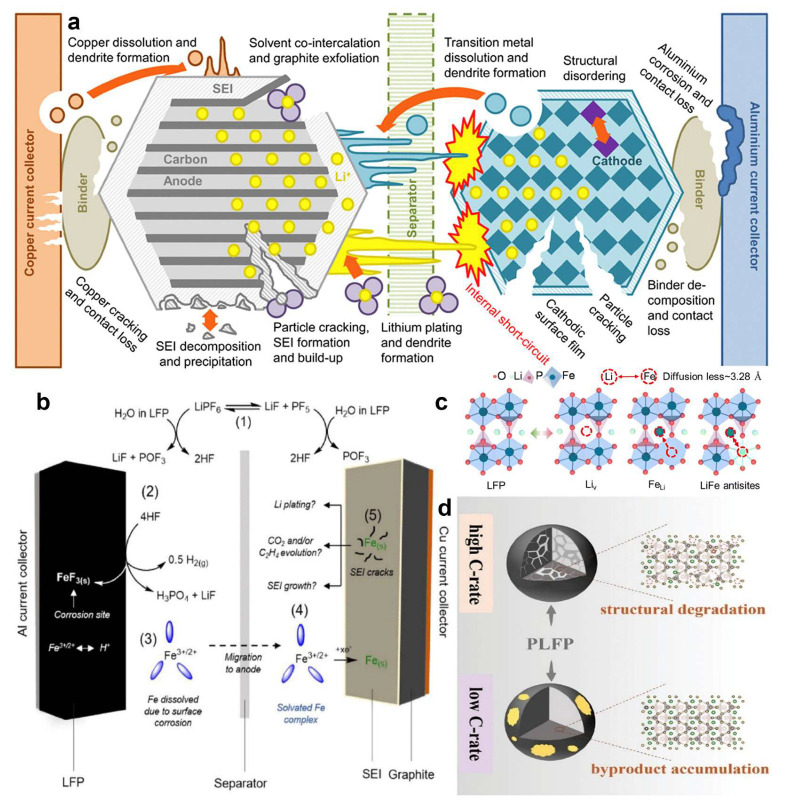
(**a**) Reveals the multi-scale failure closed loop of a lithium-ion battery from the anode through the separator to the cathode [18]. Copyright 2016 Elsevier. (**b**) The mechanism of iron dissolution [38], Copyright 2020 The Electrochemical Society. (**c**) Li–Fe antisite defects [51], Copyright 2024 Royal Society of Chemistry. (**d**) failure modes at various C-rates [47]. Copyright 2025 Royal Society of Wiley. These figures are not from the same system.

LIBs exhibit a complex array of interdependent degradation pathways that span the anode, cathode, separator, electrolyte, and interfacial regions [18]. These failure mechanisms not only compromise electrochemical performance but also pose significant safety hazards [19]. A schematic representation of these cascading degradation phenomena is illustrated in Figure 1a. On the anode side, solvent co-intercalation and repeated solid electrolyte interphase (SEI) fracture induce progressive exfoliation of graphite, particularly under high polarization conditions [20,21,22,23]. This promotes the nucleation and growth of lithium dendrites, which can penetrate the separator and cause internal short circuits. Simultaneously, copper current collectors are susceptible to electrochemical dissolution under low state-of-charge, releasing Cu^2+^ ions that migrate through the electrolyte and redeposit on the cathode surface. These metallic deposits form conductive bridges, jeopardizing battery safety and accelerating failure [24,25,26].

The separator, especially when lacking mechanical robustness or electrochemical resistance, can be breached by dendrites, further escalating short circuit risks. Meanwhile, the layered cathodes exhibit lattice collapse and oxygen evolution upon over-delithiation, leading to severe thermal and structural instability. This reciprocal degradation is further complicated by the dissolution of transition metals from the cathode, which migrate to the anode and undergo reductive deposition, catalyzing SEI deterioration. Additional contributors include binder decomposition, current collector corrosion, and mechanical disintegration of active materials. These concurrent failures ultimately converge in thermal runaway, initiated and accelerated by exothermic interfacial reactions, oxygen release, and internal electrical shorting [16].

Among all battery components, the cathode has emerged as a critical initiator of degradation, prompting significant research efforts toward its structural optimization and regenerative recycling [27,28,29,30]. In the case of LFP cathodes, five dominant degradation mechanisms are generally recognized: (1) Electrolyte decomposition: hydrolysis of the commonly used LiPF_6_ salt yields hydrofluoric acid (HF), lithium fluoride (LiF), and phosphoryl fluoride (POF_3_), especially in the presence of residual moisture and protons generated during electrolyte oxidation [31,32,33,34]. These by-products initiate chemical attack on both electrolyte and electrode surfaces; (2) HF-Induced LFP surface corrosion: although less documented than in layered oxide systems [35,36,37], HF reacts with LFP in a 4:1 molar ratio, producing gaseous H_2_, LiF, phosphoric acid (H_3_PO_4_), and insoluble iron fluoride (FeF_3_). This reaction corrodes the LFP surface and facilitates proton/Fe^2+^ exchange within the olivine lattice, weakening structural integrity [38,39,40]; (3) Fe dissolution and solvation: the corroded Fe species, particularly Fe^2+^ and Fe^3+^, undergo solvation via ligand coordination (e.g., β-diketone-type chelation), which enables Fe ion migration through the electrolyte [41,42]; (4) Anode-side Fe deposition: upon arrival at the anode, the Fe^2+^/Fe^3+^ ions are electrochemically reduced to metallic Fe nanoparticles, which embed within the SEI. These inclusions disturb the local electrochemical environment, alter SEI morphology, and act as heterogeneous nucleation sites for lithium plating [43]; (5) Catalytic SEI decomposition: the embedded Fe particles catalyze parasitic redox reactions within the SEI [44,45,46], including the decomposition of organic carbonate components, leading to gas evolution (e.g., CO_2_, C_2_H_4_), excessive SEI thickening, and heightened interfacial impedance (Figure 1b) [38,47].

Furthermore, intrinsic antisite disorder—manifesting as Li^+^/Fe^2+^ site exchanges in the LFP lattice—continues to limit capacity utilization and cycling stability. Recent studies have demonstrated that high-temperature shock (HTS) treatments can reversibly reconfigure this disorder by inducing ultrafast Li–Fe cation migration. Subjecting LFP to millisecond-scale thermal pulses enables lattice-wide hopping events that restore cationic order and enhance Li^+^ diffusion, structural stability, and electrochemical performance (Figure 1c,d) [48,49,50,51].

### 2.2. Current Collector Separation Strategies

Efficient separation of active cathode materials from aluminum (Al) current collectors is a pivotal step in LIB recycling, with profound implications for downstream purification, material regeneration, and economic viability. Conventional methods struggle with the strong adhesion induced by polyvinylidene fluoride (PVDF) binders and surface oxidation of Al. Consequently, several advanced techniques have been developed to overcome these interfacial challenges. Among them, electrochemical hydrogen bubbling, ultrasound-assisted cavitation, and chemical passivation have demonstrated notable potential for high-yield, low-energy delamination.

**Figure 2:**
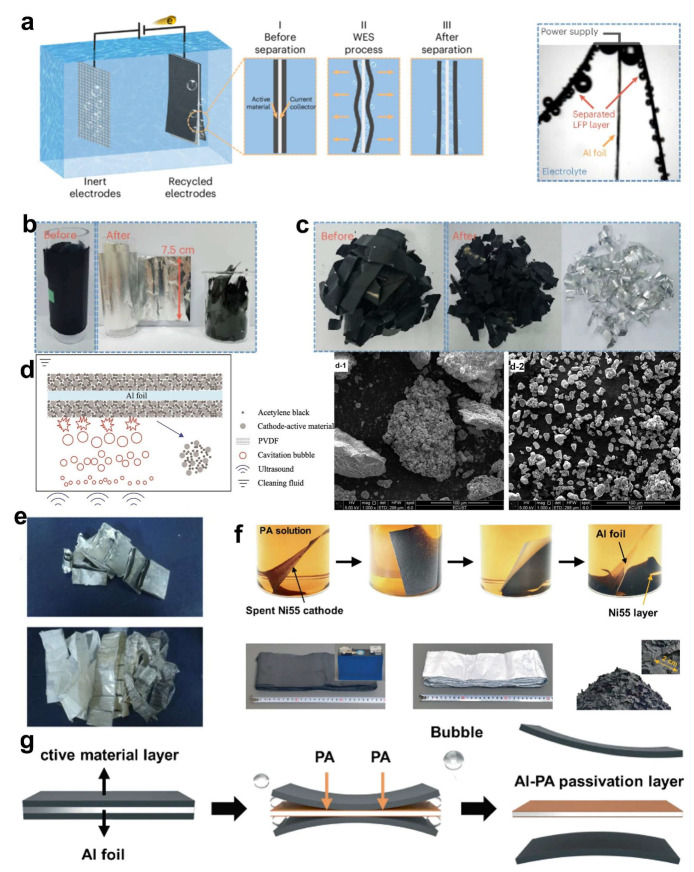
(**a**) A schematic of the HER WES process and a cross-sectional side-view image of a LiFePO_4_ electrode immersed in electrolyte after WES, with its upper side connected to the power supply and numerous bubbles visible on the electrode surface, (**b**,**c**) photographs of LFP electrodes before and after the WES treatment [52]. Copyright 2025 Nature; SEM image of the pristine LFP battery (**d-1**) and after ultrasonic delamination (**d-2**), (**e**) the aluminum foil after ultrasonic separation [53]. Copyright 2021 Elsevier; (**f**) Digital images showing the separation of the Al-foil–Ni55 layer in PA solution and photographs of the cathode active material before and after separation, (**g**) a schematic illustration of the process by which active material separates from aluminum foil in PA solution [55]. Copyright 2023 Nature.

Recently, Yang et al. [52] proposed a water-electrolysis-induced separation (WES) technique that harnesses in situ hydrogen gas evolution to delaminate the electrode layer. By immersing spent LFP or graphite electrodes in a 0.5 M (NH_4_)_2_SO_4_ electrolyte and applying a constant current, nanoscopic hydrogen bubbles form rapidly at the Al interface. These bubbles coalesce and lift the electrode coating within seconds (Figure 2a–c). The WES method achieves near-complete delamination efficiencies—99.5% for LFP in 34 s and for graphite in 3 s—while maintaining minimal contamination levels (<40 ppm) and low energy input (11 kJ kg^−1^ for LFP, 1.1 kJ kg^−1^ for graphite) [52].

An ultrasound-assisted delamination approach is proposed by Yang et al. [52] to facilitate the separation of cathode materials from Al substrates. Yang et al. [52] introduced an ultrasonic delamination method that couples PVDF binder dissolution with cavitation-induced mechanical agitation. When operated in N-methyl-2-pyrrolidone (NMP) at 70 °C under 240 W ultrasound for 90 min, this technique achieves a delamination efficiency of ~99%. Acoustic cavitation generates alternating pressure cycles that form and collapse microbubbles, releasing localized energy that disrupts the electrode/foil interface and promotes layer detachment (Figure 2d,e). The recovered cathode materials exhibit low agglomeration, facilitating efficient leaching and regeneration [53].

Moreover, Sun et al. [54] developed a bio-inspired approach using phytic acid (PA), a plant-derived organic acid, to selectively corrode Al foils. The multiple phosphate and hydroxyl groups in PA react with Al to form hydrogen gas and Al^3+^ ions, weakening the adhesive interaction with PVDF and promoting physical detachment of the cathode layer (Figure 2f,g). Additionally, PA forms a self-limiting aluminum–phytate complex that passivates the Al surface and halts further corrosion. This mechanism enables centimeter-scale recovery of intact cathode films via simple sedimentation, while reducing Al loss and minimizing downstream purification requirements [55].

The integration of advanced failure mechanism analysis with innovative cathode separation strategies forms the basis for next-generation LIB recycling. Understanding the root causes of degradation—especially in LFP-based cathodes—facilitates the rational design of rejuvenation and reuse pathways. In parallel, scalable and energy-efficient delamination techniques such as WES, ultrasonic cavitation, and PA-induced passivation represent powerful tools for sustainable resource recovery. These developments collectively pave the way for a closed-loop battery circular economy that couples environmental responsibility with high-value material utilization.

## 3. Regeneration/Refurbishment of LFP Electrodes

Regeneration of spent LFP cathodes, which aims to restore both their crystal integrity and electrochemical performance without complete decomposition into elemental constituents, has emerged as a promising low-energy and sustainable recycling approach. Recent studies have demonstrated the effectiveness of both organic lithium salts and redox-assisted methods in directly healing the structural and chemical degradation commonly observed in aged LFP cathodes.

### 3.1. Pyro-Regeneration of Spent LFP Cathodes

**Figure 3:**
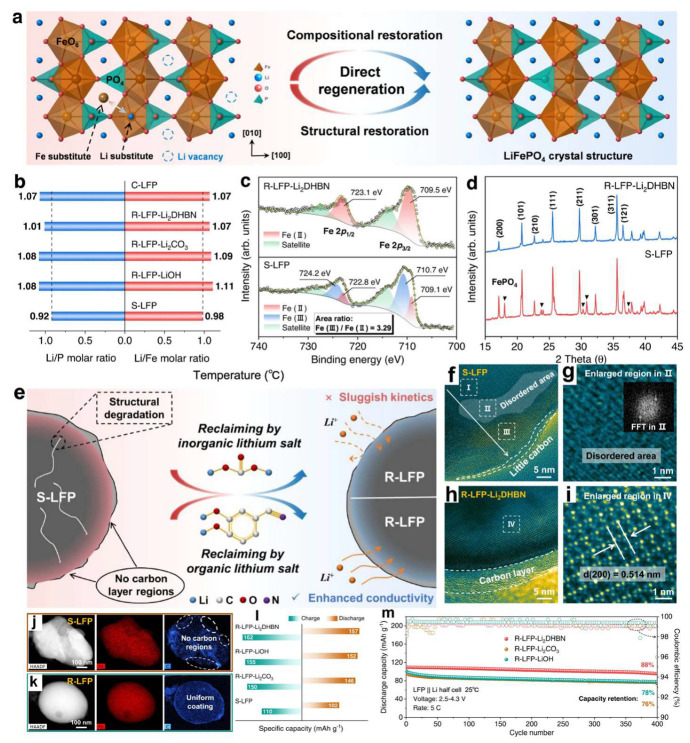
(**a**) Schematic illustration of the degraded and restored crystal structures, (**b**) Li/P and Li/Fe molar ratios determined by ICP-OES, (**c**) Fe 2p XPS spectra of S-LFP and R-LFP-Li_2_DHBN, (**d**) XRD patterns of S-LFP and R-LFP-Li_2_DHBN, (**e**) schematic of the S-LFP regeneration mechanism using inorganic and organic lithium salts, (**f**) TEM image, (**g**) HRTEM image, (**h**) TEM image, (**i**) HRTEM image, (**j**) HAADF-STEM image and EDS maps of S-LFP, (**k**) HAADF-STEM image and EDS maps of R-LFP-Li_2_DHBN, (**l**) residual and recovered capacity of R-LFP-Li_2_DHBN at 0.1 C, (**m**) long-term cycling performance at 5 C [56]. Copyright 2023 Nature.

Organic lithium salts have emerged as multifunctional reagents for regenerating spent LFP materials. Recently, Liang et al. [56] pioneered the use of a chelating organic lithium salt, 3,4-dihydroxybenzonitrile dilithium (Li_2_DHBN), to simultaneously address Li depletion and Fe(III) phase formation in spent LFP (Figure 3a,e). The regeneration involved mechanically blending Li_2_DHBN with degraded LFP, followed by sintering at 800 °C in an Ar/H_2_ atmosphere. Compared with traditional inorganic lithium sources such as Li_2_CO_3_ or LiOH, the regenerated LFP (R-LFP) exhibited restored lithium stoichiometry and significantly enhanced electrochemical performance (Figure 3b). XPS analysis confirmed near-complete reduction of Fe(III) to electrochemically active Fe(II), while structure images revealed well-aligned lattice fringes tightly encapsulated by a carbon layer derived from Li_2_DHBN pyrolysis. This in situ-formed conductive carbon shell not only improved electronic conductivity but also stabilized the electrode–electrolyte interface, yielding a capacity retention of 88% after 400 cycles at 5 °C [56].

X-ray photoelectron spectroscopy (XPS) analysis confirmed the near-complete elimination of Fe(III) species in R-LFP, highlighting the efficacy of the direct regeneration process (Figure 3c). The surface of regenerated LFP was also uniformly encapsulated by an amorphous carbon coating (Figure 3d), contributing to improved structural integrity and long-term cycling performance(Figure 3f–i). Compared with inorganic lithium salts such as Li_2_CO_3_ and LiOH, the R-LFP prepared using Li_2_DHBN exhibited superior electrochemical performance. At a current density of 0.1 C (1 C = 170 mA g^−1^), the discharge capacity of R-LFP-Li_2_DHBN reached 157 mAh g^−1^, significantly outperforming those regenerated using Li_2_CO_3_ (146 mAh g^−1^) and LiOH (152 mAh g^−1^), and much higher than the degraded LFP (102 mAh g^−1^). Remarkably, R-LFP-Li_2_DHBN maintained a capacity of 110 mAh g^−1^ after 400 cycles at 5 C, corresponding to 88% capacity retention (Figure 3m), attributable to the protective and conductive carbon shell formed during organic lithium salt pyrolysis (Figure 3j,k). These results underscore the dual functionality of organic lithium salts in restoring Li stoichiometry and Fe(III) valency, while simultaneously forming conductive carbon networks to stabilize regenerated LFP particles. This strategy provides a new perspective for the direct regeneration of aged LFP cathodes with both chemical and structural degradation.

**Figure 4:**
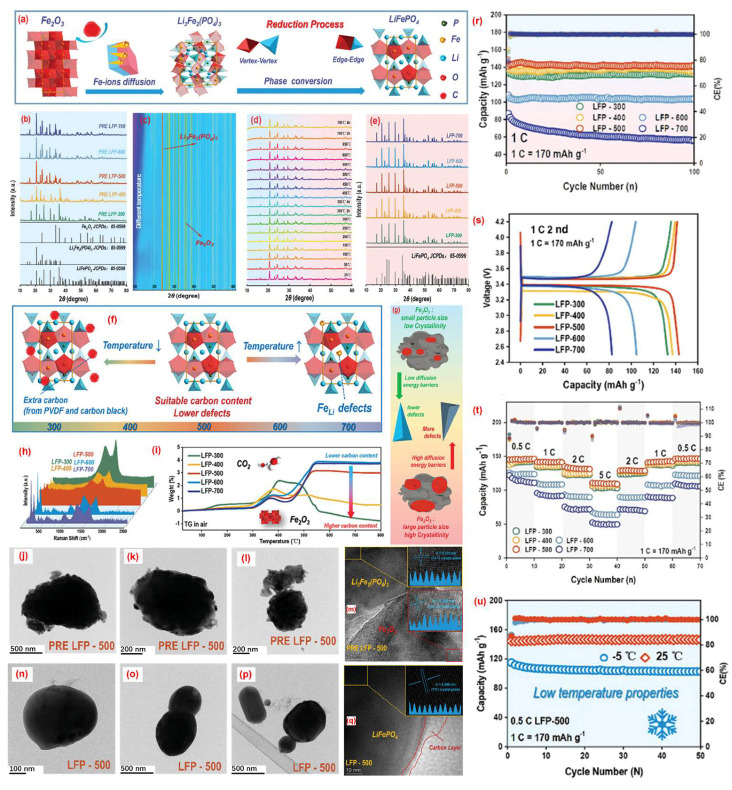
(**a**) Stage-wise transformation mechanism from the precursor to LiFePO_4_ sample, (**b**) XRD of the precursor, (**c**,**d**) in situ XRD of pure PRE LFP-500 (glucose-free), (**e**) XRD of the regenerated LFP sample, (**f**) structural diagrams of different recycled samples, (**g**) defect formation mechanism in recycled samples, (**h**) Raman spectra, (**i**) TGA curves, (**j**–**m**) TEM and HRTEM images of PRE LFP-500, (**n**–**q**) TEM and HRTEM images of LFP-500, (**r**) cycling performance at 1 C, (**s**) charge–discharge curves of recycled samples at 1.0 °C, (**t**) rate performance of recycled samples at various current densities, (**u**) low-temperature cycling performance of LFP-500 [57]. Copyright 2023 Wiley.

To address heterogeneous degradation and poor active site accessibility, Ge et al. [57] employed a redox-mediated regeneration method to rejuvenate degraded LFP cathodes, addressing issues such as non-uniform degradation, varied particle sizes, and inaccessible active sites. Their approach involved a two-step process: oxidation of spent LFP at controlled temperatures in air to remove residual carbon and binder materials, followed by reductive annealing with glucose as a carbon precursor and reducing agent. Specifically, pre-oxidized LFP (PRE-LFP) was ball-milled with glucose in a 5:0.75 mass ratio at 400 rad for 2 h, dried, and subsequently annealed at 700 °C for 10 h under N_2_ atmosphere (Figure 4a). X-ray diffraction analysis showed that, following annealing, the regenerated LFP exhibited a pure olivine phase without detectable impurities (Figure 4e), and the evolution of Fe_2_O_3_ and Li_3_Fe_2_(PO_4_)_3_ peaks reflected the differences in crystallinity of intermediate phases (Figure 4b,c).

The optimal oxidation temperature was identified as 500 °C. Below this temperature, residual carbon layers persisted on the surface of degraded LFP particles (Figure 4h,i), while higher temperatures led to excessive Fe and Li loss, generating crystal defects and compromising lattice integrity (Figure 4f). TEM analysis revealed that PRE-LFP-500 featured irregular surface textures and lattice damage (Figure 4j–m), whereas the fully regenerated LFP displayed smooth surfaces uniformly coated with an amorphous carbon shell derived from glucose pyrolysis (Figure 4n–q). Electrochemical tests confirmed that the sample oxidized at 500 °C and reduced with glucose (LFP-500) exhibited the best rate capability and cycling stability among all tested conditions (Figure 4r–u). The superior performance was attributed to improved lattice ordering and surface carbon encapsulation, which jointly enhanced lithium ion diffusion and electron transport (Figure 4q) [57].

Similarly, Liu et al. [58] reported a direct regeneration strategy for spent LFP cathodes via a thiourea-assisted solid-state sintering process. In this method, the spent cathode material was mixed with LiOH and thiourea, followed by sintering at 800 °C for 6 h under an Ar/H_2_ atmosphere. Thiourea played a dual role in the regeneration process: the amino groups (–NH_2_) preferentially coordinated with Fe ions, enabling targeted repair of antisite defects and inactive FePO_4_ phases through stronger Fe^3+^–N interactions compared to Fe^2+^–N. Additionally, the thermal decomposition products of thiourea created a mildly reductive environment during high-temperature treatment, which effectively suppressed particle agglomeration. The regenerated LFP (R-LFP) exhibited excellent electrochemical performance under various charge–discharge rates [58]. Notably, the R-LFP-1 sample delivered outstanding rate capability and long-term cycling stability, maintaining a capacity of 108 mAh g^−1^ after 900 cycles at 3 C with a capacity retention of 84%.

Zhang et al. [54] adopted an all-solid route to re-synthesize LiFePO_4_/C electrodes, employing a simple heat-treatment process to purify and homogenize the recovered cathode material. Neither waste acids nor alkaline liquids are used, thereby avoiding secondary environmental pollution. They further investigated the influence of Li_2_CO_3_ content on the performance of the regenerated LiFePO_4_/C material and successfully re-synthesized a high-purity, high-tap-density LiFePO_4_/C product. The route is green, offers high recovery yields, and is low-cost. The re-synthesized LiFePO_4_/C material exhibits outstanding overall electrochemical performance [54].

Chen et al. [59] innovatively proposed an in-situ advanced oxidative metallurgy technique that selectively extracts lithium from LiFePO_4_ via Fenton oxidation instead of conventional metallurgical processes. Lithium can be completely released without disrupting the olivine structure of LiFePO_4_, leaving FePO_4_ as a precursor. The oxidation of Fe(II) and the liberation of Li^+^ in LiFePO_4_ are predominantly triggered by the rapid attack of abundant •OH radicals generated during the advanced oxidation process. The released Li^+^ is recovered as Li_2_CO_3_ and recombined with FePO_4_ to re-manufacture LiFePO_4_ [59].

Collectively, these findings demonstrate that both organic lithium salt-assisted and redox-mediated direct regeneration approaches can effectively restore the Li content, suppress Fe(III) formation, and reconstruct the structural integrity of degraded LFP cathodes. Importantly, in situ formation of conductive carbon shells during thermal treatment plays a critical role in improving cycling stability and electrochemical kinetics. These strategies offer scalable, low-energy pathways for the sustainable reuse of LFP cathodes and open new avenues for high-value, direct recycling of lithium iron phosphate batteries.

### 3.2. Hydro-Regeneration of Spent LFP Cathodes

**Figure 5:**
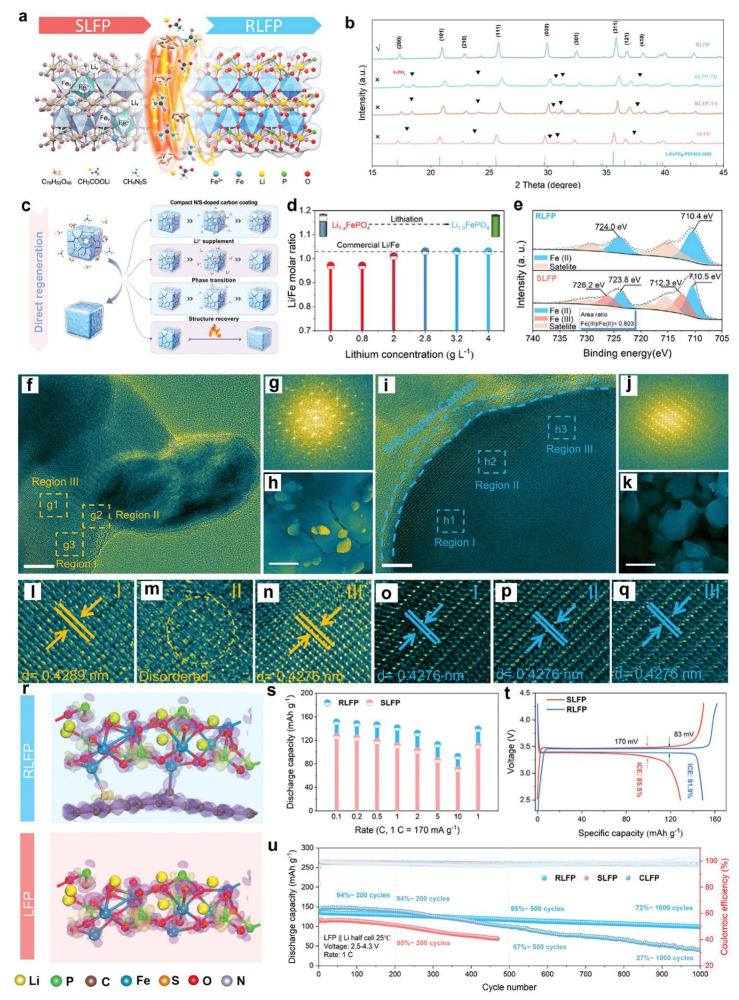
(**a**) Schematic of the SLFP recycling principle and direct regeneration strategy mechanism, (**b**) XRD patterns of SLFP, RLFP-TA, RLFP-TU, and RLFP, (**c**) schematic of the SLFP regeneration mechanism, (**d**) Li/Fe molar ratios of RLFP at different Li concentrations, (**e**) Fe 2p XPS spectra of SLFP and RLFP, (**f**,**g**) HRTEM images of SLFP (scale bar = 10 nm), (**h**) SEM image of SLFP (scale bar = 500 nm), (**i**,**j**) HRTEM images and corresponding FFT patterns of RLFP (scale bar = 10 nm), (**k**) SEM image of SLFP (scale bar = 500 nm), (**l**–**n**) HRTEM images of the regions marked by dashed rectangles in (**f**), (**o**–**q**) HRTEM images of the regions marked by dashed rectangles in (**i**), (**r**) differential charge distribution models of LFP and RLFP, (**s**) rate performance of SLFP and RLFP, (**t**) initial charge–discharge curves of SLFP and RLFP at 0.1 C, (**u**) cycling performance of SLFP, RLFP, and CLFP at 1 C [60]. Copyright 2024 Wiley.

In an effort to address the limitations of conventional recycling strategies—namely, low economic returns and environmental concerns—Cao et al. [60] developed a direct regeneration approach for spent LFP (SLFP) cathodes through the synergistic application of tannic acid (TA) and thiourea (TU) [60]. This strategy not only targets the critical antisite Li/Fe defects that commonly impair Li^+^ diffusion kinetics in degraded LFP but also constructs a multifunctional heteroatom-doped carbon interface under relatively mild conditions, thereby enhancing the physicochemical stability and electrochemical performance of the regenerated LFP (RLFP). Furthermore, theoretical simulations based on differential charge density models revealed that the N/S-doped carbon shell in RLFP facilitates surface charge delocalization through the formation of C–Fe and S–Fe coordination bonds (Figure 5r), thereby enhancing both electronic conductivity and Li^+^ transport kinetics [61,62].

In a complementary strategy, Song et al. [63] developed a practical hydrothermal strategy for regenerating industrial black powder derived from spent LiFePO_4_ (LFP) cathodes (denoted as S-BM) using glycerol as a multifunctional reductant (Figure 5a). Glycerol, rich in hydroxyl groups, acts as an effective electron donor [63,64,65], reducing Fe(III) to Fe(II) and repairing Fe–Li antisite defects (Fe_Li_). In their approach, the S-BM was mixed with LiOH and glycerol and subjected to hydrothermal treatment to form a precursor (R-BM-1), which was subsequently annealed at 500–600 °C under an Ar atmosphere for 3 h to yield regenerated LFP (R-BM). The chelating ability of glycerol, combined with its compatibility with fractured particles, suppressed Ostwald ripening and promoted grain-boundary reconnection, resulting in uniform layered microcrystals (Figure 5e,f) [66,67]. Simple post-annealing effectively restored the particle morphology and crystalline structure (Figure 5b–d), while the regenerated material exhibited recovery of Fe–O bonding [68], mitigating structural distortion and enhancing Li^+^ diffusion kinetics. As a result, the R-BM cathode delivered a high discharge capacity of 123.2 mAh g^−1^ at 5 C after 500 cycles with a capacity retention of 93.1% (Figure 5g), along with excellent rate capability (Figure 5h), demonstrating its industrial scalability and electrochemical robustness.

To tackle surface-level degradation, Li et al. [69] proposed a near-surface reconstruction method based on a multifunctional lithium acetylacetonate (Liacac) solution to repair degraded phases in aged LFP cathodes [69]. The acetylacetonate anions (Acac^−^) in the Liacac solution exhibit strong chelating affinity toward Fe(III), facilitating controlled dissolution of amorphous by-products and restructuring of the near-surface region [70,71]. This chelation-driven process effectively removes detrimental species such as disordered phases, residual fluorides, and cathode–electrolyte interphase (CEI)-derived impurities. Simultaneously, Liacac serves as both a coordination modulator and a localized lithium source, compensating for lithium loss in the cathode. The reconstructed surface enables direct Li–O connectivity and lowers Li^+^ diffusion barriers, significantly improving reinsertion kinetics. The regenerated cathodes showed enhanced lithium diffusion kinetics and maintained 88.5% capacity after 400 cycles at 1 C, offering a low-temperature, environmentally friendly regeneration route.

Direct regeneration of spent LFP cathodes presents a viable path toward the sustainable and value-added recycling of lithium-ion batteries. The strategies discussed herein—including organic salt-assisted sintering, redox-mediated treatments, multifunctional carbon precursor utilization, and chelation-driven surface reconstruction—demonstrate high efficacy in restoring Li stoichiometry, reversing Fe(III) formation, and improving electronic conductivity via in situ carbon encapsulation. These approaches not only enable the retention of original cathode structure but also enhance electrochemical durability, thus providing a promising framework for scalable and eco-friendly LFP battery recycling.

## 4. Upcycling Spent LFP Batteries as Functional Materials

With the rapid growth of LFP-based cathode consumption, sustainable recycling strategies that go beyond elemental recovery are attracting increasing attention. Recent advances have demonstrated the potential to directly convert spent LFP electrodes into high-performance catalysts, particularly for electrochemical water splitting and environmental remediation applications. These upcycling approaches not only mitigate solid waste concerns but also significantly enhance the functional value of the recovered materials.

### 4.1. Upcycling for Electrochemical Water Splitting

**Figure 6:**
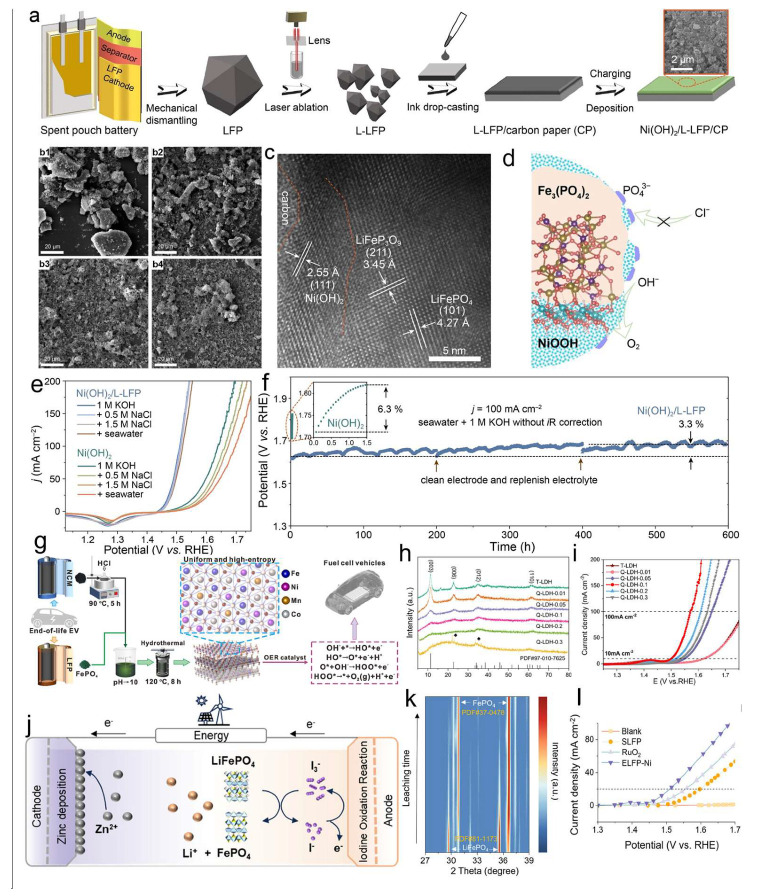
(**a**) Schematic of the Ni(OH)_2_/L-LFP synthesis process. SEM images of L-LFP: as-prepared sample (**b1**); after 15 min laser ablation (**b2**); after 60 min laser ablation (**b3**); after 120 min laser ablation (**b4**). (**c**) high-resolution TEM image of Ni(OH)_2_/L-LFP, (**d**) Cl^−^-repulsion mechanism during seawater oxidation of Ni(OH)_2_/L-LFP [72]. Copyright 2024 Wiley.; (**e**) LSV curves of Ni(OH)_2_/L-LFP and Ni(OH)_2_ in saline and alkaline seawater, (**f**) chronoamperometric potential curves of Ni(OH)_2_ and Ni(OH)_2_/L-LFP in alkaline seawater, (**g**) general schematic of LDH catalyst synthesis from spent lithium-ion battery cathodes, (**h**) XRD patterns of the spent cathodes, (**i**) LSV curves of T-LDH and Q-LDHs in 1 M KOH [81]. Copyright 2025 Wiley.; (**j**) Schematic of Li^+^ leaching from LFP using I_3_^−^/I^−^ redox mediator, (**k**) XRD patterns of LiFePO_4_ during leaching, (**l**) LSV curves of the ELFP-Ni catalyst prepared from Li-extracted LiFePO_4_ compared with blank, SLFP, and RuO_2_ [82]. Copyright 2025 Wiley.

Lee et al. [72] developed a laser-fragmentation-enabled upcycling approach to address the degradation of catalyst performance in chloride-rich seawater environments (Figure 6a) [73,74,75,76,77,78]. In this strategy, bulk LFP waste was disintegrated into micro-structured fragments via high-energy laser ablation, As can be seen, after laser ablation for 15 min (Figure 6(b2)), 60 min (Figure 6(b3)), and 120 min (Figure 6(b4)), the particle size is significantly reduced compared with the original sample (Figure 6(b1)), forming a reconstructed L-LFP structure that interfaces with surface-deposited Ni(OH)_2_ to generate a NiOOH/Fe_3_(PO_4_)_2_ heterojunction (Figure 6c). This heterointerface was found to significantly enhance the selective oxidation of Cl^−^ (Figure 6d), while simultaneously promoting OH^−^ adsorption and electron transfer during the OER process. The in situ formation of NiOOH and Fe_3_(PO_4_)_2_ creates interfacial electric fields that modulate the local charge environment, facilitating intermediate adsorption and lowering the thermodynamic barriers of OER [79,80]. As a result, this catalyst demonstrates outstanding Cl^−^-resistant activity and durability in alkaline seawater, achieving a low overpotential of 237 mV at 10 mA cm^−2^, and retaining 96.7% of its activity after 600 h at 100 mA cm^−2^ (Figure 6e,f), highlighting its practical potential for large-scale saline water electrolysis [72].

Meantime, Yao et al. [81] further advanced the concept by integrating dual-source waste valorization. Using a co-leaching and co-precipitation route (Figure 6g), they simultaneously upcycled spent LFP and NCM cathodes to synthesize quaternary layered double hydroxides (Q-LDHs), denoted as Q-LDH-0.1 (Figure 6h). The resulting catalyst exhibited high OER performance, attributed to the uniform incorporation of Fe, Ni, Co, and Mn atoms, which introduced a highly disordered coordination environment and mixed-valence states. These features collectively enhanced the electronic interactions and adsorption dynamics of OER intermediates. Q-LDH-0.1 required only 270 mV overpotential to deliver a current density of 10 mA cm^−2^ (Figure 6i). This dual-waste valorization strategy provides a scalable and cost-effective pathway for transforming multiple cathode wastes into value-added OER catalysts [81].

Besides, Zhou et al. [82] proposed an iodine-mediated electrochemical extraction strategy to simultaneously recover lithium and upgrade delithiated LFP into OER catalysts. Utilizing a recyclable I_3_^−^/I^−^ redox couple in a one-sided liquid-phase reaction system (Figure 6j), lithium was extracted with high leaching efficiency (93%) and recovered as Li_2_CO_3_, while Zn metal was simultaneously electrodeposited for direct use in Zn–air batteries or hydrogen production. The delithiated FePO_4_ residue was directly repurposed as an active OER catalyst. In situ XRD confirmed the structural evolution from LiFePO_4_ to FePO_4_ (Figure 6k), and the catalyst achieved an overpotential of only 250 mV at 10 mA cm^−2^, outperforming commercial RuO_2_ (Figure 6l). This integrated process not only simplifies the separation steps but also enables closed-loop resource recovery with dual valorization routes [82].

### 4.2. Upcycling for NO Reduction

**Figure 7:**
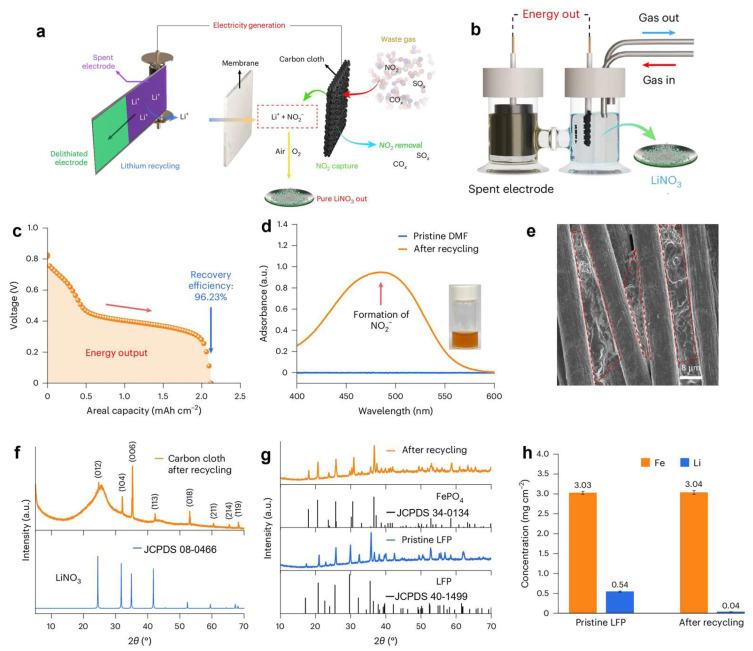
(**a**) Integrated lithium recovery and NOₓ capture system, (**b**) high-capacity recovery operation 10 × 3 cm^2^ LFP electrode placed in an H-cell), (**c**) electrical output curve during recovery at a current density of 0.1 mA cm^−2^, (**d**) UV–Vis spectrum of the recovered product before oxidation, (**e**) SEM image of the carbon cloth after recovery with the red box highlighting the formed solid LiNO_3_ product, (**f**) XRD pattern of the recovered product, (**g**) XRD patterns of the LFP electrode before and after recovery, (**h**) changes in lithium content of the LFP electrode before and after recovery [83]. Copyright 2025 Nature.

More catalytic applications beyond OER have also been explored based on recycled LFP electrodes. For example, Wang et al. [83] developed a waste-free electrochemical lithium extraction technique that simultaneously captures nitrogen monoxide (NO) from industrial flue gas while generating both electrical energy and high-purity lithium nitrate (LiNO_3_, > 99%) as the end product (Figure 7a). This process operates without the need for excessive chemical reagents or external energy input, achieving a lithium recovery efficiency of up to 97% and delivering an energy output of 66 Wh per kilogram of processed electrode material [83]. The spontaneous extraction of Li^+^ from spent LFP cathodes is coupled with the two-electron reduction of NO into nitrite (NO_2_^−^), which subsequently reacts with Li^+^ to form lithium nitrite (LiNO_2_). Upon exposure to air, LiNO_2_ is oxidized into stable LiNO_3_ [84,85], releasing significant electrical energy in the process (Figure 7b). The energy output profile exhibits a distinct plateau at approximately 0.4 V (Figure 7c), while the UV–vis absorbance spectrum reveals a broad peak around 500 nm, indicative of NO_2_ generation (Figure 7d). Solid products deposited on carbon fiber substrates contain N and O elements (Figure 7e), and X-ray diffraction (XRD) analysis confirms the exclusive formation of phase-pure LiNO_3_ without detectable impurities (Figure 7f–h). Notably, the residual Li content in delithiated FePO_4_ is minimal, measured at only 0.04 mg cm^−2^, indicating nearly complete lithium extraction.

### 4.3. Upcycling for Photocatalytic Degradation of Tetracycline

**Figure 8:**
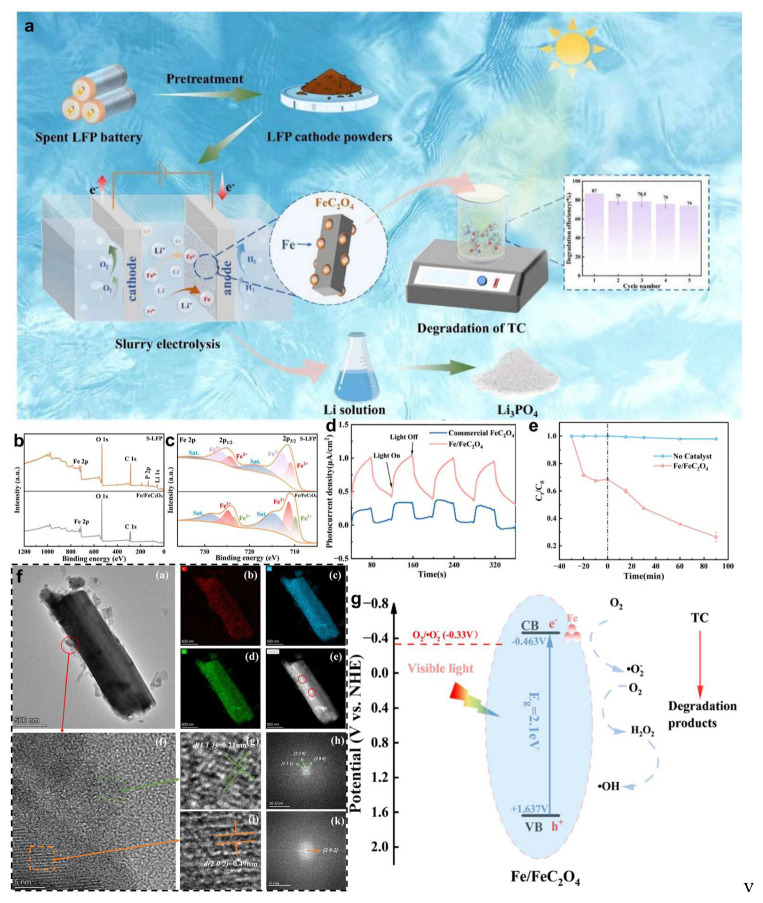
(**a**) Schematic of LFP recovery coupled with photocatalysis, (**b**) XPS survey spectra of S-LFP and Fe/FeC_2_O_4_, (**c**) high-resolution Fe 2p XPS spectra, (**d**) transient photocurrent response, (**e**) TC degradation curves using Fe/FeC_2_O_4_ versus without catalyst, (**f**)-a: the SEM image of Fe/FeC_2_O_4_; b–e: the corresponding elemental mapping images; f: the high-resolution image of Fe/FeC_2_O_4_; g,i: magnified high-resolution images of different regions; h–k: the FFT patterns of the respective regions, (**g**) proposed photocatalytic mechanism for TC degradation under visible-light irradiation by Fe/FeC_2_O_4_ [86]. Copyright 2025, Elsevier.

Complementarily, Xue et al. [86] introduced a slurry-electrolysis approach for LFP recovery that enables efficient separation of lithium and iron components (Figure 8a), while converting the extracted iron into high-value Fe/FeC-O_4_ composites with excellent photocatalytic properties [86]. In this process, a mixed electrolyte composed of oxalic acid (0.6 M) and sodium sulfate is employed, enabling nearly 100% lithium recovery from the LFP cathode powder, which is dispersed directly in the solution. The system utilizes RuO_2_/Ti mesh and stainless steel as cathode and anode, respectively, and is operated under magnetic stirring to promote controlled electrolysis. Post-electrolysis, magnetic separation is used to isolate free iron species, leading to the formation of Fe/FeC_2_O_4_ (Figure 8b) [87]. X-ray photoelectron spectroscopy (XPS) confirms the presence of metallic iron species (Figure 8c), which are also evident as edge domains in the TEM images of the rod-like FeC_2_O_4_ nanostructures (Figure 8f). Owing to its narrow bandgap and strong visible-light absorption, FeC_2_O_4_ effectively generates electron-hole pairs under LED illumination, where photogenerated electrons reduce dissolved oxygen to produce reactive O_2_^−^ species that drive the degradation of organic pollutants [88,89]. As a result, the material achieves 87% tetracycline (TC) degradation within 120 min (Figure 8d,e), and retains 74% efficiency after five cycles, highlighting its outstanding photocatalytic stability and reusability [86].

### 4.4. Upcycling for Ferricyanide-Assisted Hydrogen Production

**Figure 9:**
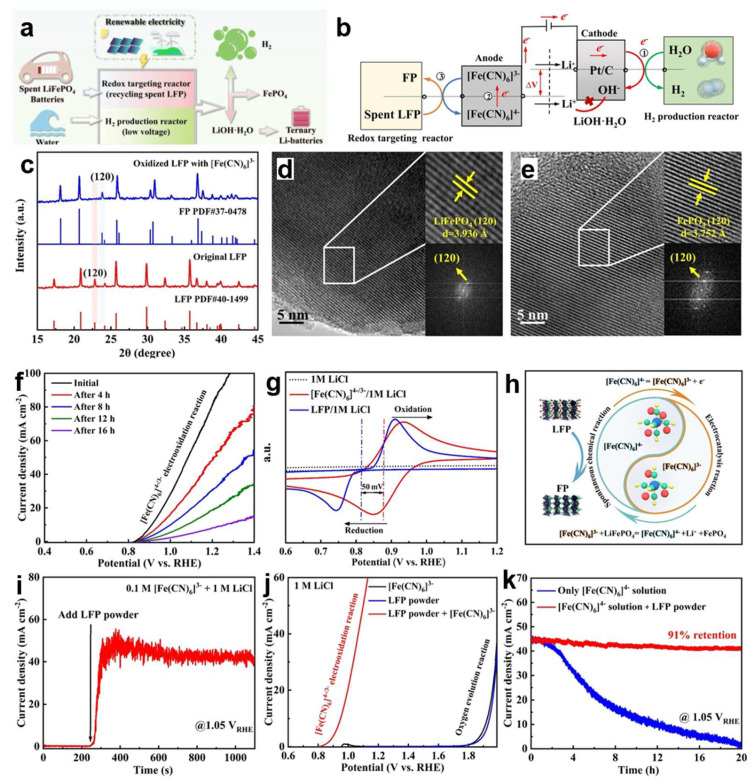
(**a**) Schematic of LFP recovery coupled with hydrogen evolution reaction, (**b**) detailed process schematic of [Fe(CN)_6_]^4−^ electro-oxidation reaction coupled HER and spent LiFePO_4_ battery recycling, (**c**) XRD patterns of pristine LFP and [Fe(CN)]-oxidized LFP, (**d**) HR-TEM image of pristine LFP, (**e**) HR-TEM image of [Fe(CN)]-oxidized LFP, (**f**) LSV curves of the carbon cloth electrode in 0.1 M [Fe(CN)] electrolyte at various scan times, (**g**) cyclic voltammograms of [Fe(CN)] and LFP in 1 M LiCl, (**h**) schematic of the [Fe(CN)_6_]^4−^/^3−^ redox cycle upon addition of LiFePO_4_ powder, (**i**) chronoamperometric current response of the carbon cloth electrode in 0.1 M [Fe(CN)_6_]^3−^ and 1 M LiCl electrolyte at 1.05 V_RHE, (**j**) LSV curves of [Fe(CN)_6_]^3−^ at the carbon cloth electrode in the absence and presence of LFP powder, (**k**) long-term stability of [Fe(CN)_6_]^4−^ electro-oxidation before and after introduction of LFP powder [90]. Copyright 2024 Wiley.

In another innovative approach, Wu et al. [90] devised an innovative hydrogen production system that couples water electrolysis with a waste-assisted redox cycle involving ferricyanide/ferrocyanide species (Figure 9a,b). This approach not only enables low-cost and energy-saving hydrogen generation but also facilitates the simultaneous valorization of spent LFP materials into high-purity lithium hydroxide monohydrate (LiOH·H_2_O) and FePO_4_, offering an environmentally benign and economically viable route for recycling end-of-life LFP batteries (Figure 9c–e). In their system, carbon cloth (CC) was used as the anode, immersed in an anodic electrolyte containing [Fe(CN)_6_]^4−^ and LFP powder. A charge-regulated ion-selective (CRIS) membrane acted as a separator to effectively prevent both solid-phase contaminants and redox species ([Fe(CN)_6_]^4−^/[Fe(CN)_6_]^3−^) from diffusing to the cathode compartment. The introduction of LFP enables the in situ regeneration of [Fe(CN)_6_]^4−^, which can then be continuously electrooxidized, forming a sustainable and self-reinforcing anodic redox loop (Figure 9f–h). Without LFP, the anodic current remains negligible, indicating the absence of effective redox cycling. However, upon the addition of LFP, a rapid redox interaction occurs between [Fe(CN)_6_]^3−^ and the LFP surface, resulting in the formation of fresh [Fe(CN)_6_]^4−^ (Figure 9i). This cyclic oxidation-reduction behavior is evidenced by the appearance of an anodic peak around 0.8 V corresponding to the oxidation of [Fe(CN)_6_]^4−^ (Figure 9j). After 20 h of continuous operation, the system maintained 91% of its initial current density, whereas in the absence of LFP, the current rapidly decayed—highlighting the critical role of LFP in sustaining long-term anodic redox activity (Figure 9k) [90].

These collective studies exemplify how spent LFP cathodes—once considered non-functional waste—can be transformed into value-added catalysts for diverse applications, including water splitting, nitrogen fixation, pollutant degradation, and hydrogen production. Through structural design, redox regulation, and interface engineering, upcycling pathways are becoming increasingly versatile, scalable, and aligned with green chemistry principles. As research continues to integrate battery recycling with catalyst design, spent LFP is poised to emerge as a cornerstone resource in the sustainable materials economy.

## 5. Elemental Recycling Toward Value-Added Products

In the quest for sustainable and value-added utilization of spent lithium iron phosphate (LFP) batteries, increasing attention has been devoted to element-specific recovery strategies that selectively reclaim lithium (Li) and phosphorus (P) while enabling their transformation into functional or marketable products. These approaches not only mitigate resource depletion and environmental risks but also align with the principles of a circular economy by reintroducing recovered elements into new value chains.

### 5.1. Selective Lithium Recovery and Conversion

**Figure 10:**
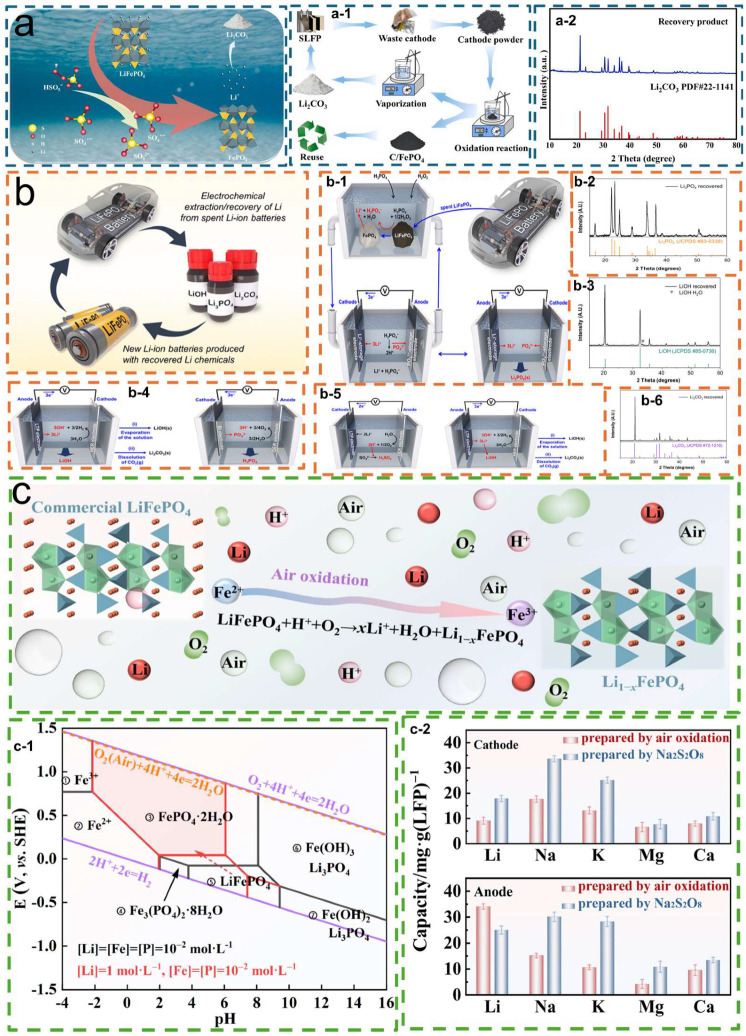
(**a**) Schematic of the oxidative leaching process for recovering spent LiFePO_4_ and Li_2_CO_3_; (**a-1**) flow chart for recovering spent LiFePO_4_ and Li_2_CO_3_ via oxidative leaching; (**a-2**) XRD patterns of the recovered Li_2_CO_3_, (**b**) electrochemical process for recovering spent LiFePO_4_, Li_2_CO_3_, LiOH, and Li_3_PO_4_ [91]. Copyright 2025 Elsevier.; (**b-1**) Schematic of Li_2_CO_3_ recovery; (**b-2**) XRD pattern of Li_3_PO_4_; (**b-3**) XRD pattern of LiOH; (**b-4**) schematic of Li_3_PO_4_ recovery; (**b-5**) schematic of LiOH recovery; (**b-6**) XRD pattern of Li_2_CO_3_ [92]. Copyright 2025 American Chemical Society.; (**c**) Schematic of the leaching mechanism; (**c-1**) thermodynamic analysis and schematic of the leaching mechanism; (**c-2**) cation contents in the cathode and anode after 90 cycles [93]. Copyright 2023 Elsevier.

Yuan et al. [91] developed a simple and efficient leaching method using peroxymonosulfate (PMS) to selectively extract Li^+^ from lithium iron phosphate (LFP) cathodes (Figure 10(a-1)). This process releases Li^+^ into the solution by oxidizing Fe^2+^, achieving a lithium leaching efficiency of up to 99.9% (Figure 10a). The extracted Li^+^ ions subsequently react with sodium carbonate (sodium carbonate) to form lithium carbonate (Li_2_CO_3_), while the residual FePO_4_ maintains the integrity of the olivine framework structure (Figure 10(a-2)). This study provides a simple and selective method for lithium extraction, showing significant advantages in process simplification, efficiency improvement, and host crystal structure preservation for potential reuse [91].

In a more integrated electrochemical method, Choi et al. [92] reported a dual-chamber system consisting of a lithium extraction unit and a lithium recovery unit, which can achieve environmentally friendly and cost-effective lithium recovery in the form of lithium phosphate, Li_2_CO_3_, or lithium hydroxide. Notably, the system can regenerate the acidic leaching agent consumed during lithium extraction, thereby minimizing chemical consumption and secondary waste generation. When phosphoric acid was used as the leaching solution (Figure 10(b-1)), lithium ions dissolved in the weakly acidic environment while iron phosphate did not, enabling the efficient separation of Li_3_PO_4_ (Figure 10(b-2)). When strong alkali (KOH) was used as the leaching solution (Figure 10(b-4)), lithium ions underwent hydrogen evolution reaction (HER) or oxygen reduction reaction (ORR) at the cathode in dilute LiOH solution, leading to the accumulation of concentrated LiOH (aq), which could be evaporated to obtain LiOH (Figure 10(b-3)). In a strong acid leaching solution under electrolysis, LiOH was generated (Figure 10(b-5)), which, after capturing CO_2_ produced Li_2_CO_3_ (Figure 10(b-6)). This closed-loop design provides a sustainable framework for lithium recovery from spent LFP batteries. The study further elaborated on electrode selection, working principles, and variations in extraction/recovery configurations, highlighting its feasibility for large-scale applications [92].

In addition, Zhu et al. [93] proposed a direct oxidative leaching strategy based on air as the oxidant, which can selectively extract lithium ions from lithium iron phosphate batteries under thermodynamic guidance. Under weakly acidic and oxidative conditions, lithium ions (Li^+^) were effectively leached (Figure 10(c-1)), while divalent iron ions (Fe^2+^) were oxidized to trivalent iron ions (Fe^3+^), and the entire process did not disrupt the stability of the olivine FePO_4_ lattice structure (Figure 10c). After cycling, significant compositional differences appeared between the cathode and anode (Figure 10(c-2)). Compared with traditional methods, this strategy exhibits higher selectivity, milder operating conditions, and lower cost advantages, making it an ideal solution for industrial-scale lithium recovery [93].

### 5.2. Phosphorus Recovery and Functional Upcycling

Beyond lithium, the valorization of phosphorus from spent LFP batteries has also gained traction, particularly for applications in sustainable agriculture. Li et al. [94] demonstrated a dual-element recovery process in which both Li and P are reclaimed, with phosphorus further converted into a P-grafted slow-release fertilizer (P-SRF) possessing acid-resistant properties. The process begins with NaOH-assisted ultrasonication of LFP, followed by oxidative delithiation using sodium persulfate (Na_2_S_2_O_8_) to recover Li. The remaining delithiated residue is then reacted with sodium thiosulfate (Na_2_S·9H_2_O), resulting in the formation of the final fertilizer product. Mechanistic studies revealed that phosphorus is recovered in the form of HPO_4_^2−^/H_2_PO_4_^−^, which effectively lowers the activation energy for urea–acrylic acid polymerization and provides a pH-buffering effect beneficial for acidic soils. XRD analysis confirmed the amorphous nature of the resulting phosphorus–urea complex, while pot experiments with maize demonstrated enhanced plant growth and nutrient uptake. This strategy not only addresses phosphorus recovery but also realizes its upcycling into a functional and commercially relevant product, particularly suitable for application in horticulture, acidic soil remediation, and ecological slope stabilization [95,96].

**Figure 11:**
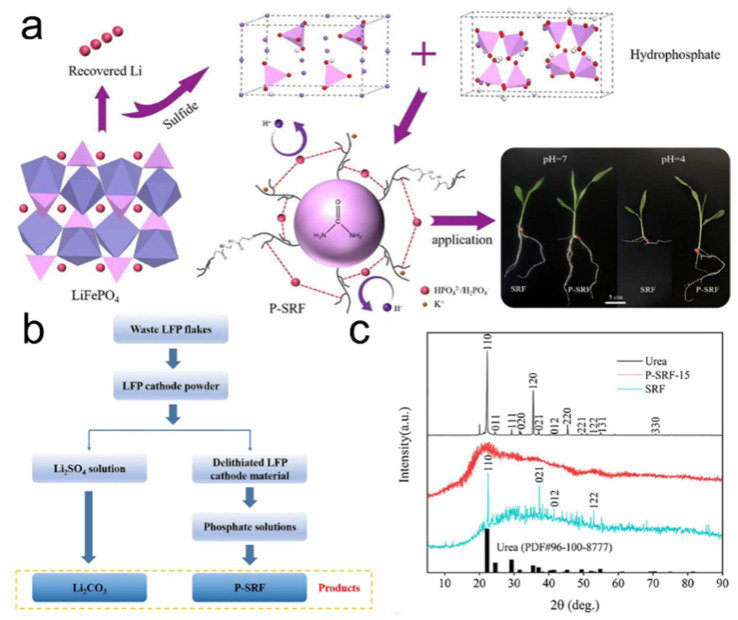
(**a**) Schematic of Li recovery from LFP and P-fertilizer production, (**b**) flowchart for P-SRF synthesis and Li recovery, (**c**) XRD patterns of urea, SRF, and P-SRF-15 [94]. Copyright 2020 Elsevier.

Therefore, these element-specific recovery routes represent a paradigm shift from traditional resource extraction to targeted, value-oriented recycling. By selectively extracting Li and P and converting them into high-purity lithium salts or functional fertilizers, these approaches extend the life-cycle of battery-derived elements and enhance the economic viability of LFP recycling processes. Moreover, the preservation of structural iron phosphates such as FePO_4_ offers additional potential for material regeneration or reuse in secondary applications (Figure 11a–c). These advancements underscore the transformative potential of integrating chemical selectivity, process efficiency, and product functionality in the upcycling of spent LFP batteries—paving the way for sustainable, closed-loop recycling ecosystems [94].

## 6. Summary and Outlook

With the exponential growth of EVs, LFP batteries have become a dominant energy storage technology due to their intrinsic safety, long cycle life, and cost-effectiveness. However, the surging number of end-of-life LFP batteries presents both environmental challenges and resource recovery opportunities. Unlike traditional pyrometallurgical and hydrometallurgical methods that focus primarily on elemental extraction, recent advances have led to the development of innovative strategies that aim to regenerate or upcycle spent LFP into functional materials with added value. This review has comprehensively summarized the failure mechanisms of LFP cathodes, including Fe^2+^/Fe^3+^ redox imbalance, Li loss, and structural degradation, and discussed how these issues motivate targeted regeneration and upcycling strategies. Current research has shown that techniques such as electrochemical delamination, ultrasonic-assisted separation, and mild chemical passivation can effectively detach cathode materials from current collectors, improving the efficiency of downstream recycling processes. Furthermore, both thermal and hydrothermal regeneration routes—assisted by organic lithium salts, redox mediators, or multifunctional chelating agents—have demonstrated remarkable potential in restoring lithium stoichiometry and healing crystal defects in degraded cathodes.

Beyond regeneration, the upcycling of spent LFP into high-value functional materials such as electrocatalysts, photocatalysts, slow-release fertilizers, and redox mediators offers a sustainable path for reusing not only lithium but also iron and phosphorus resources. Notably, laser fragmentation, heterojunction construction, and electrochemical delithiation have enabled the transformation of aged cathodes into active materials for seawater splitting, nitrogen fixation, and pollutant degradation. These upcycling approaches represent a paradigm shift toward circular economy principles by extending the functional lifespan of battery-derived materials. Table 1 presents the advantages of the various recycling methods.

Despite these promising developments, several critical challenges remain:

(1) Scalability and standardization: most current regeneration and upcycling techniques remain confined to laboratory-scale demonstrations. The lack of standardized processes and industrial scalability hinders commercialization; (2) Economic viability: the relatively low intrinsic value of Fe and P in LFP cathodes limits the economic attractiveness of recovery compared to cobalt- and nickel-rich systems. Cost-effective and energy-efficient processes are urgently needed; (3) Material complexity and heterogeneity: variations in LFP battery composition, degradation levels, and contaminant profiles complicate batch processing and reduce the reproducibility of recycling outcomes; (4) Life-cycle and environmental assessment: while many proposed methods show excellent technical performance, their long-term environmental impacts and carbon footprints are still underexplored, highlighting the need for comprehensive life-cycle assessment and techno-economic analysis.

**Table 1:** Advantages of different recycling approaches.

Technology	Methods	Advantages	Reference
Separation of the current collector	Water-dissociation-induced separation (WES) technology	Fast stripping speed with minimal pollution	[52]
	Ultrasound-assisted cavitation exfoliation	Rapid delamination with excellent structural integrity of the recovered materials	[53]
	Chemical passivation method	Simple operation, scalable for large-scale use	[55]
LFP regeneration and repair	Chelating organolithium salt-assisted repair	The surface carbon layer significantly enhances both conductivity and stability.	[56]
	Redox-mediated regeneration	Enables more complete regeneration of LFP	[57]
	Thiourea-assisted solid-state sintering process	Effectively suppresses particle agglomeration	[58]
	Synergistic repair using tannic acid (TA) and thiourea (TU)	C–Fe and S–Fe coordination bonds promote surface charge delocalization, markedly enhancing ionic conductivity	[60]
	Direct regeneration of spent LiFePO_4_ with glycerol	Effectively promotes interfacial reconnection and restores the layered micro-crystalline structure	[63]
	Multifunctional lithium acetylacetonate (Li(acac))-assisted repair	During repair, it efficiently reconfigures the amorphous by-products.	[69]
Upcycling of LFP	Upcycling via electrochemical water splitting	Upgraded into a green, clean-energy catalyst for hydrogen production	[81,82]
	Upcycling for nitrogen-oxide reduction recycling	When LFP is upcycled into LiNO_3_, it simultaneously captures nitrogen oxides from flue gas.	[83]
	Upcycling for tetracycline photocatalytic degradation	Upcycled into an economical and environmentally friendly photocatalyst	[86]
	Upcycling for ferricyanide-assisted hydrogen production	Reduces the cost of hydrogen production while simultaneously recovering LFP into LiOH.	[90]
Element recovery	Simple and efficient selective Li leaching with peroxymonosulfate (PMS)	The residual FePO_4_ retains the original olivine structural framework.	[91]
	Dual-chamber system for Li^+^ extraction and Li^+^ recovery units	One chamber consumes the leachate while the other electrochemically reduces it, drastically reducing chemical reagent use and pollution.	[92]
	Direct air-oxidative leaching	High selectivity—only Li is recovered	[93]
	Dual-element recovery of Li and P for the production of phosphorus fertilizer	Converting spent LFP into cost-effective phosphorus fertilizer	[95]

According to these issues, future research should focus on the following directions to unlock the full potential of LFP recycling and upcycling: (1) Development of integrated recycling platforms: designing modular, closed-loop systems that combine material separation, direct regeneration, and upcycling functions will enhance process efficiency and reduce waste; (2) Mechanistic understanding and data-driven optimization: advanced characterization techniques, such as in situ spectroscopy, synchrotron-based imaging, and machine learning-driven process optimization, can accelerate the rational design of regeneration pathways; (3) Policy and regulatory support: establishing clear regulatory frameworks and incentive mechanisms will promote the industrial adoption of green LFP recycling technologies, especially in regions with high EV penetration; (4) Co-valorization of multiple waste streams: exploring synergistic recovery strategies that integrate spent LFP with other cathode materials (e.g., NCM) or industrial by-products could maximize resource utilization and economic return.

In conclusion, the sustainable recycling and upcycling of spent LFP batteries represent a multifaceted and evolving field with significant environmental, economic, and technological implications. As the global demand for lithium-ion batteries continues to rise, collaborative efforts across disciplines—spanning materials science, electrochemistry, environmental engineering, and policy—will be essential to establish a robust and circular battery economy that harnesses the full value of spent LFP materials.
